# Spatio-temporal patterns of attacks on human and economic losses from wildlife in Chitwan National Park, Nepal

**DOI:** 10.1371/journal.pone.0195373

**Published:** 2018-04-19

**Authors:** Babu Ram Lamichhane, Gerard A. Persoon, Herwig Leirs, Shashank Poudel, Naresh Subedi, Chiranjibi Prasad Pokheral, Santosh Bhattarai, Bishnu Prasad Thapaliya, Hans H. de Iongh

**Affiliations:** 1 Institute of Cultural Anthropology and Development Sociology, Faculty of Social and Behavioural Sciences, Leiden University, Leiden, Netherlands; 2 Evolutionary Ecology Group, Faculty of Sciences, University of Antwerp, Campus Drie Eiken, Antwerp, Belgium; 3 National Trust for Nature Conservation (NTNC), Khumaltar, Lalitpur, Nepal; 4 Department of National Parks and Wildlife Conservation (DNPWC), Kathmandu, Nepal; 5 Institute of Environmental Sciences (CML), Faculty of Sciences, Leiden University, Leiden, Netherlands; Universita degli Studi di Sassari, ITALY

## Abstract

Wildlife attacks on humans and economic losses often result in reduced support of local communities for wildlife conservation. Information on spatial and temporal patterns of such losses in the highly affected areas contribute in designing and implementing effective mitigation measures. We analyzed the loss of humans, livestock and property caused by wildlife during 1998 to 2016, using victim family’s reports to Chitwan National Park authorities and Buffer Zone User Committees. A total of 4,014 incidents were recorded including attacks on humans, livestock depredation, property damage and crop raiding caused by 12 wildlife species. In total >400,000 US dollar was paid to the victim families as a relief over the whole period. Most of the attacks on humans were caused by rhino, sloth bear, tiger, elephant, wild boar and leopard. A significantly higher number of conflict incidents caused by rhino and elephant were observed during full moon periods. An increase in the wildlife population did not coincide with an equal rise in conflict incidents reported. Underprivileged ethnic communities were attacked by wildlife more frequently than expected. Number of attacks on humans by carnivores and herbivores did not differ significantly. An insignificant decreasing trend of wildlife attacks on humans and livestock was observed with significant variation over the years. Tiger and leopard caused >90% of livestock depredation. Tigers killed both large (cattle and buffalo) and medium sized (goat, sheep, pig) livestock but leopard mostly killed medium sized livestock. Most (87%) of the livestock killing during 2012–2016 occurred within the stall but close (<500m) to the forest edge. Both the percentage of households with livestock and average holding has decreased over the years in buffer zone. Decreased forest dependency as well as conflict mitigation measures (electric and mesh wire fences) have contributed to keep the conflict incidents in control. Strengthening mitigation measures like construction of electric or mesh wire fences and predator-proof livestock corrals along with educating local communities about wildlife behavior and timely management of problem animals (man-eater tiger, rage elephant etc.) will contribute to reduce the conflict.

## Introduction

With ongoing fragmentation and degradation of the remaining natural areas [1], wildlife species are forced to live in close proximity to humans leading to frequent human-wildlife interactions [2]. Such interaction is more intense in the areas where large mammals like Asian elephants (*Elephas maximus)*, greater one-horned rhinoceros (*Rhinoceros unicornis*), Bengal tigers (*Panthera tigtris tigrris)* and common leopards (*Panthera pardus ficusa)* are in high densities [3–4] in relatively small protected areas within human dominated landscapes [5]. Attacks on humans and property damage by wildlife and subsequent persecution of wildlife in retaliation are generally referred to as ‘human-wildlife conflict’ [6–7]. This is a frequent phenomenon especially in the fringe of protected areas and forests [8–9]. Prevention or mitigation of such negative interaction is challenging when multiple endangered species of conservation significance are involved [10].

We selected Chitwan National Park (CNP) in Nepal for this study because it has been experiencing frequent and intensive human-wildlife conflicts since its establishment in 1973 [11]. CNP is also a flagship park in Nepal whose success or failure largely determines the overall direction of wildlife conservation in the country [12]. Conservation was started in core areas of the park in 1970s through strict protection by the national army with limited rights of people. As a consequence, park-people (human-human) conflict was more pronounced in the initial years of park establishment [11, 13–14]. Soon, the need for support of local communities living in the vicinity of the park was recognized to sustain the conservation [15]. Participatory conservation programs were initiated in the early 1990s in Nepal [16]. The government endorsed a Buffer Zone Policy in 1996 with a provision of 30–50% of the park revenue diverted to the respective buffer zone [17]. Following these participatory conservation initiatives, habitat restoration in the buffer zone, especially in community forests, created opportunities to both wildlife and people. Strict protection by the army in the core area also continued. During the past four decades, as a result of these conservation efforts, CNP has observed a gradual increase in the large mammal populations [3–4]. The park has high density of mega-herbivores such as elephants and rhinos [4] and large carnivores like tigers and leopards [3, 18]. In the surrounding areas of the Park, human density is also relatively high (261.5 persons per km^2^ in 2011) and the human population is increasing at 2.1% annually [19]. Probably as a consequence, a rise in the number of conflict incidents from wildlife has been reported by previous studies, especially in the buffer zone areas [8–9, 20]. In contrast, another study from Chitwan also showed that human and wildlife (tiger as example) can co-exist with temporal displacement in well protected areas at fine spatial scale [12].

Studies in Africa show the effect of moon phase on the activity of carnivores, especially lions [21–23], herbivores [24–25] and their interaction with humans. Packer et al. [22] from their study in Tanzania found more attacks by lions on humans during the dark nights following the full moon, when the moon rises more than an hour after dusk. Cozzi et al. [21] reported no difference in activity of lion and hyena over the lunar cycle but found an influence of moonlight availability on the hunting behavior of wild dog and cheetah. Crop-raiding by African elephants was less during the full moon phase [25]. We are not aware of published studies on the impact of moon-phase on human-wildlife conflict in Asia.

Previous studies about human-wildlife interaction in CNP and BZ focused on either a single species [8, 20, 26] or only on human casualty [9–10] but comprehensive analysis of human-wildlife conflicts over a longer time-span remain un-reported. Thus, in our study, we present a comprehensive analysis of human-wildlife conflicts around CNP during a time span of 18 years (1998 to 2016) using the largest available dataset for a park in Nepal. We analyzed the types of loss from wildlife in time and space, and factors associated. We tested two hypotheses in our study: 1) Lower number of conflict-incidents occur during full-moon phases and 2) human-wildlife conflict incidents increase with the increase in the wildlife population.

## Materials and methods

### Study area

Chitwan National Park (CNP) (27°16.56’– 27°42.14’N and 83°50.23’– 84°46.25’E; area 953 km^2^), a World Heritage Site, is Nepal’s first National Park established in 1973. It is a part of the Terai Arc Landscape, a priority tiger conservation landscape [5]. The park has a monsoon dominated sub-tropical climate with an average monthly maximum temperature between 24°C– 38°C, monthly minimum temperature between 11°C– 26°C, annual rainfall ~ 2250 mm and relative humidity 89–98% (2000–2010). The park is well known for its biodiversity with a species diversity of approximately 70 mammals, over 600 birds, 56 reptiles and amphibians, 156 butterflies, 120 fish [27]. It is also one of the core breeding sites of tigers [3]. CNP holds the world’s second largest population of greater one-horned rhinoceros [28].

**Fig 1:**
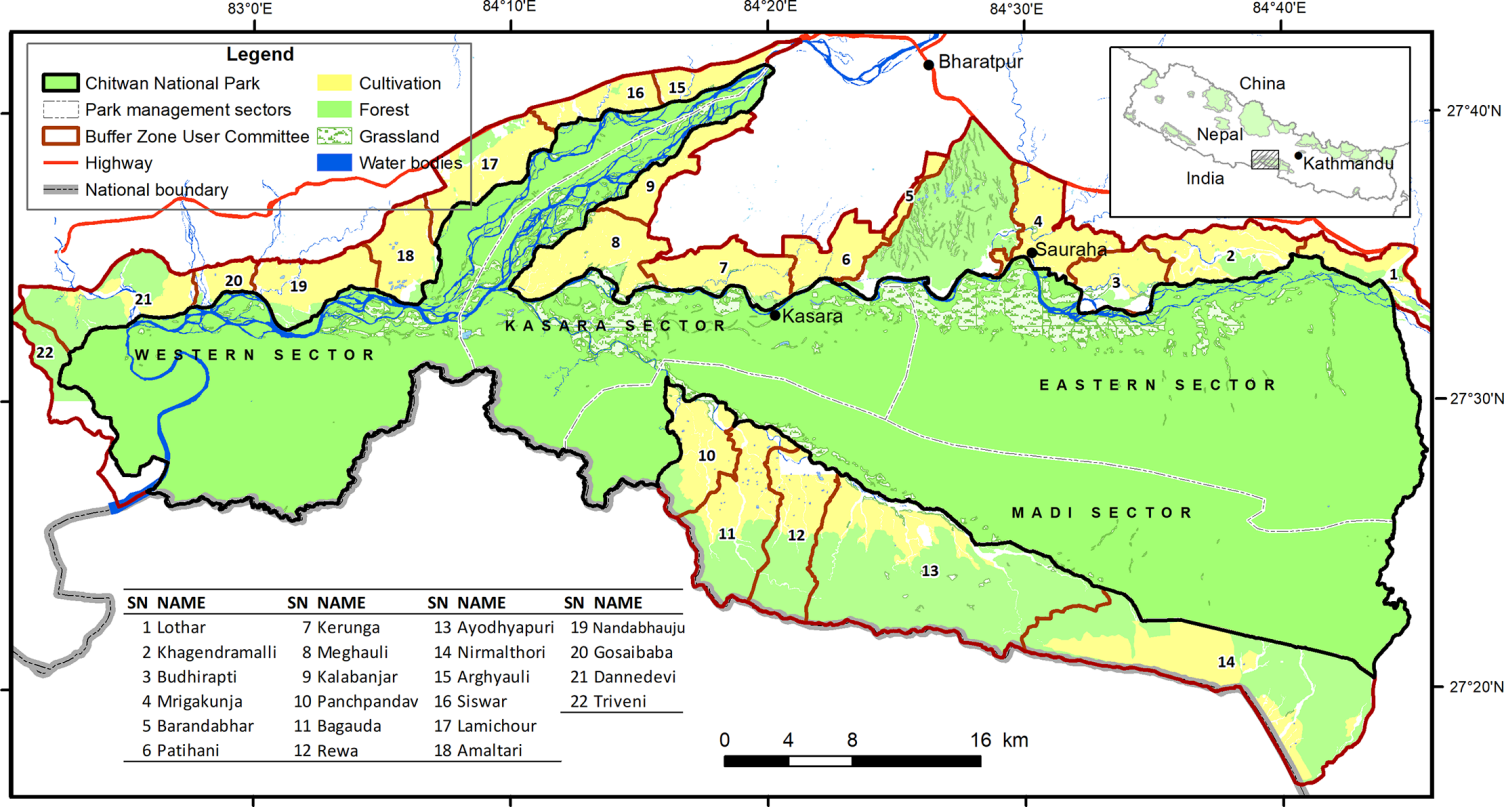
Chitwan National Park and buffer zone area, showing the land cover and management sectors. The labels (1–22) represents the Buffer Zone User Committees (BZUC) and the table (top left) gives the names of respective BZUC.

The park is dominated by forest (80%) including majority of sal (*Sorea robusta*) forest followed by riverine forest and mixed hardwood forest. In addition, there are grasslands (12%), exposed surface (5%) and water bodies (3%) [18]. The park is drained by three major river systems, i.e., Narayani, Rapti and Reu rivers. The Narayani River marks the western boundary, the Rapti River marks the northern boundary, Reu River and the international border with India along the Valmiki Tiger Reserve marks the southern boundary (Fig 1). The Parsa National Park is contagious in eastern boundary. A corridor forest called Barandabhar connects park with the northern hill forest (Fig 1).

An additional 750 km^2^ of buffer zone (BZ) surrounding CNP (~ 5 km) was created in 1996 (21 Km^2^ of BZ was included into core area in 2016). More than half (55%) of the BZ consists of wildlife habitat such as forests, grasslands, shrub land, river and water bodies; the rest the area is used for agriculture and settlements [3]. There are >80 community forests in the buffer zone which are managed by the communities. The BZ includes > 45,000 households in 12 municipalities from four districts (Chitwan, Makawanpur, Nawalparasi and Parsa) [19]. There are ~1700 user groups under 22 Buffer Zone User Committees (BZUC) which are administered by a Buffer Zone Management Committee at central level [29]. Historically there were only a few settlements of the indigenous *Tharu*, *Bote* and *Darai* communities surrounding the Park. Many people from the hilly area migrated into the Chitwan Valley after the eradication of malaria in the mid-1950s [4]. Now the community is mixture of indigenous people and ‘Hills migrants’ (*Brahmin*, *Chhetries*), ‘Ethnic migrants’ (*Tamang*, *Gurung*, *Magar* etc.), ‘Dalit’ or so called untouchables (*Kami*, *Damai*, *Sarki* etc.) and other minorities (*Madhesi*, *Muslim* etc.) [19]. Primarily people depend on subsistence agriculture although many new economic activities such as tourism and commercial farming are increasing. Livestock keeping is an integral part of subsistence agriculture, and grazing was common in the buffer zone till early 2000s but it shifted swiftly towards stall feeding.

### Loss from wildlife reported to park and buffer zone authorities

We collected data on wildlife attacks on humans and economic loss reported to the CNP authorities and the BZUC during 1998 to 2016. People started to report the loss from wildlife (primarily attacks to human and livestock depredation) to the BZUCs after the relief scheme for wildlife victims started in 1999 along with the implementation of the Buffer Zone Program [17, 29]. A guideline for relief distribution was endorsed by a meeting of the Buffer Zone Management Committee in 1999 [29]. The wildlife victims in the BZ self-reported the incidents through applications to the local authorities (CNP or BZUC) primarily to claim compensation (only partial cost is covered so it is termed ‘relief’ hereafter). The conflict incidents were verified by the BZUC and subsequently relief was released as per the guidelines. These data of relief application and distribution were kept in registers by BZUCs between 1998 and 2009. Government endorsed the relief guideline of wildlife losses in 2009 and designated respective protected areas or district forest offices for relief distribution [10]. Thus, CNP started to process and verify the relief applications as from 2009 onwards. Since then, the government revised the guideline two times (in 2013 & 2015) increasing the relief amounts [29]. We compiled all the relief applications of wildlife victims reported to both BZUCs and CNP during 18 years (1998 to 2016). The data were managed according to Nepalese fiscal year which runs from mid-July to mid-July based on the Nepalese Calendar (Bikram Sambat). For the consistency of the data for time series analysis, we used these fiscal years. Data of initial years (1998/99 to 2006/07) included victim’s name and address, respective BZUC, fiscal year, type of loss, wildlife causing the loss, amount claimed and received. Data after 2006/07 also include the date of the incident [29].

### Detailed data collection of livestock depredation

We visited 254 households who lost livestock in last five years (2012–2016) to verify the compensation claim records and get additional information about the incidents. The field survey was conducted during March–May 2016 and February–March 2017. Name and address of the applicants were obtained from the database of CNP & BZUCs. Household head or family member (above the age of 16) was interviewed using a pre-structured questionnaire (S1 File). The research and the questionnaire was approved by the ethics committee of the Institute of Cultural Anthropology and Development Sociology. Similarly. the study was also approved by the 'Technical Committee' of Department of National Parks and Wildlife Conservation which issues the research permits to studies in protected areas in Nepal. We obtained written consent of the interviewee before starting the interview. We have anonymized the identity of the interviewee before proceeding to analysis. All the necessary approvals have been obtained from the Government authorities and buffer zone user committees. GPS location of the house and livestock depredation place were recorded. Socio-economic status of the family, livestock herding practices, preventive measures and relief for the loss were collected in a standard format. We digitized the forest edge (border of the forest and cultivated areas) using high-resolution satellite images in Google Earth.

### Data analysis and statistics

We categorized the data into four types of losses a) attacks on humans (death & injury), b) house and property loss, c) livestock depredation (buffalo, cattle, goat, sheep, pig, duck/chicken) and d) crop raiding. Based on surname of the victim we derived the ethnicity of the victim into five categories of having different livelihood strategies– 1) *Hill migrant* (Brahmin, Chhetri and Thakuri migrated from hills), 2) *Ethnic migrant* (Ethnic communities of hills like Gurung, Magar, Tamang, Newar etc. migrated to Chitwan), 3) *Indigenous Terai* (Tharu, Bote, Darai, Mushahar), 4) *Dalit* (under-privileged casts of Kami, Damai, Sarki etc.) and 5) Others (Madhesi, Mushlim etc.). The surname of a person is a reliable indicator of the ethnicity in Nepal [30].

We also assigned the lunar day (1 –new moon, 15 –full moon) for the date of incident using the Gregorian-Lunar Calendar Conversion Table of Hong Kong Observatory (http://www.hko.gov.hk/gts/time/conversion.htm). We defined six moon phases of five-day period blocks. For instance a ‘new moon’ phase was defined as the period from two days prior to new moon to two days after new moon [24]. A similar five-day block was used for new moon and other four moon phases in between.

The conflict incidents were associated to the spatial layer of BZUC in Q-GIS [31] based on the address of the victim for spatial analysis. Descriptive summaries of yearly, monthly and seasonal wildlife attacks on humans and livestock depredation were calculated using Pivot table function of Microsoft Excel 2013 (Microsoft Redmond, USA) and Statistical analysis were done in R [32]. Chi-square tests of independence were applied to compare frequency of attacks (death and injury) and livestock depredation by wildlife species over the years, seasons, months and moon phases. An independent t-test was applied to compare the incidents caused by herbivores and carnivores, human death and injuries, and livestock depredation by tiger and leopard. Shapiro-Wilk test was performed to check normality of the data. We also performed a Pearson’s correlation test between livestock depredation frequency and the number of people on foreign employment from Chitwan district over the years as a measure of livelihood change [19, 33]. Foreign employment is one of the major factors in Nepal to reduce forest dependency with shortage of labor for grazing and other agricultural work as well as adopt alternative livelihood with increased capital [34]. The distance between the livestock depredation location and nearest forest edge and park boundary was calculated in QGIS using NNJoin plugin [31].

We used a linear regression to test the hypothesis that the frequency of conflict incidents increases respective to an increasing wildlife population. Frequency of human attacks by tiger and rhino over the years during the study period was modeled as a function of the tiger and rhino population. Data on the tiger and rhino population in CNP & BZ over the years (2000–2015) were collected from published reports of the surveys in different years [3–4, 28, 35–36]. The surveys were done within 3–5 years interval. The population for the years in between the surveys was reconstructed using a linear regression.

## Results

### Types of incidents & relief payment

A total of 4,014 incidents of human and economic loss from 12 wildlife species (Table 1) were reported to BZUCs or CNP authority during 18 years (1998 July to 2016 July) including 732 attacks on humans (168 fatalities and 564 injury), 2213 incidents of livestock depredation, 418 incidents of damage to house and property and 651 crop raiding incidents.

**Table 1:** Types of loss caused by wildlife in buffer zone of Chitwan National Park. Number in the parenthesis indicates the frequency of reported cases of the incident caused by the particular wildlife species.

Species	Attacks to Human	House & property loss	Livestock depredation	Crop raiding*
Blue bull*	death (1), injury (1)	-	-	-
Spotted deer*	injury (1)			paddy (2)
**Elephant**	death (26), injury 33)	house damage (301), grain storage (83) compound wall, toilet, water tank etc. (11), vehicle (3)	-	paddy (328), maize (17), wheat (2) banana (1), others (20)
**Gaur**	injury (3)	-	-	-
**Leopard**	injury (36)	-	buffalo calf (9), cattle calf (18), goat (550), sheep (8), pig (46), duck/chicken (2)	
**Mugger crocodile**	death (1)**, injury (2)	-	cattle (1), goat (4)	-
**Burmese python**	-	-	duck/chicken (4)	-
**Rhino**	death (55), injury (180)	crop storage (4)	-	paddy (123), wheat (110), banana (2)sugarcane (5), others (25)
Sambar deer*	injury (1)	-	-	-
**Sloth bear**	death (5), injury (142)	-	goat (67), pig (4)	-
**Tiger**	death (64), injury (55)	-	buffalo (189), cattle (362), goat (718), pig (42), sheep (14)	-
Wild boar*	death (2), injury (41)	-	-	paddy (3), others (1)

* Compensation scheme covers the crop raiding by elephant, rhino and wild water buffalo. Although crop raiding by deer and wild boar is widespread, it is not reported by the locals.

**There is a case of a human killed by mugger crocodile inside the park in 2016.

**Fig 2:**
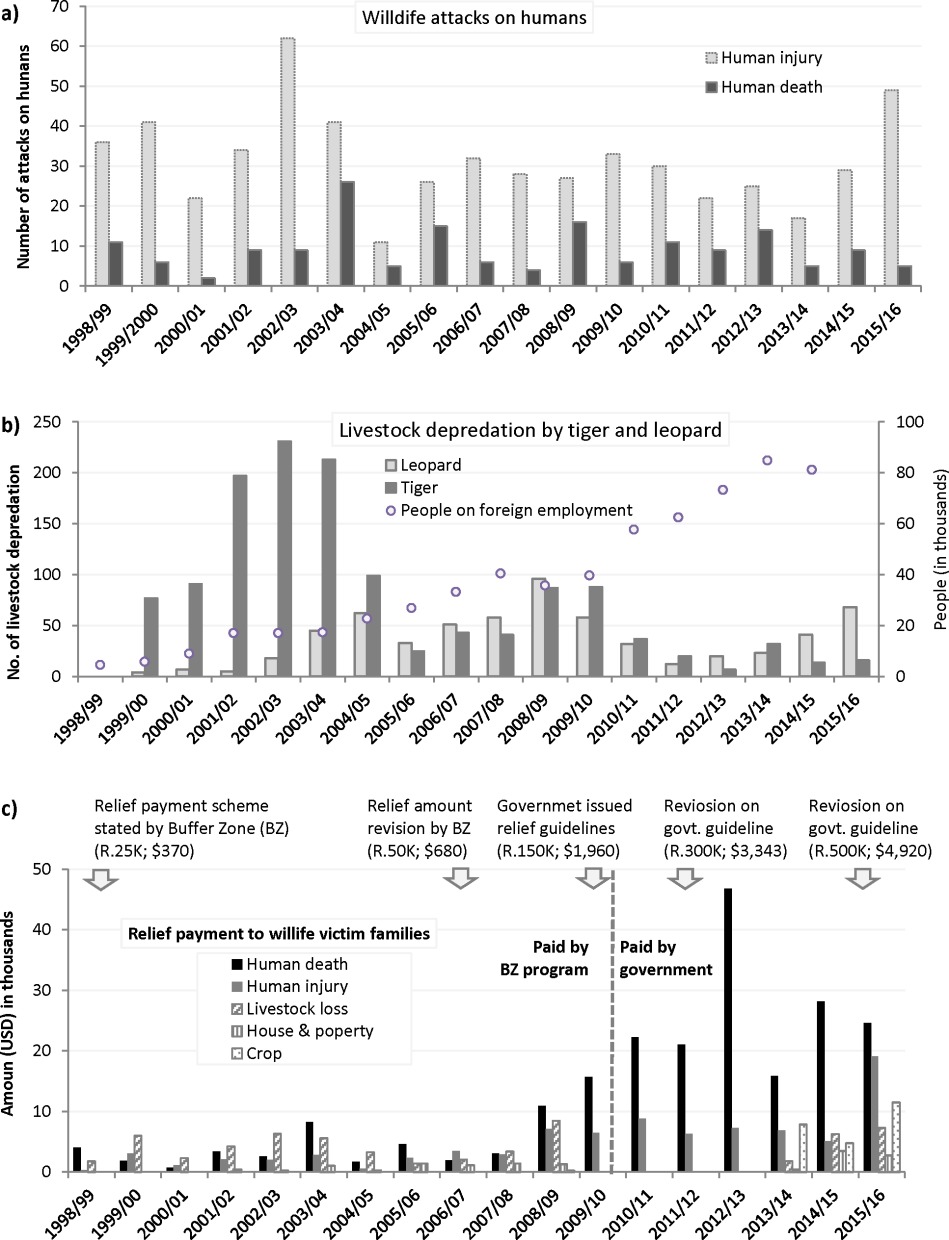
Wildlife attacks on humans, livestock depredation and relief payments over the years in Buffer Zone of Chitwan National Park, Nepal, a) Human death and injury b) livestock depredation caused by tiger and leopard, and its relation with people on foreign employment c) Amount of relief distribution to the victim families with timeline of relief distribution scheme. The numbers in parenthesis is the relief amount per victim of human death provisioned in relief guidelines of Buffer Zone or government, R = Nepalese Rupees, K = thousand.

A total of USD 403,648.51 (Nepalese Rupees 33,911,971) was paid as relief to the victims’ families for wildlife attacks or economic loss from wildlife during 1998–2016 (S1 Table). A majority (54%) of the payments was provided to families as a relief for a relative who died in a wildlife attack, followed by treatment of injured ones (21.5%), relief for livestock depredation (13.8%), crop raiding (7.1%) and property loss (3.5%) (Fig 2).

### Effect of moon phase

**Fig 3:**
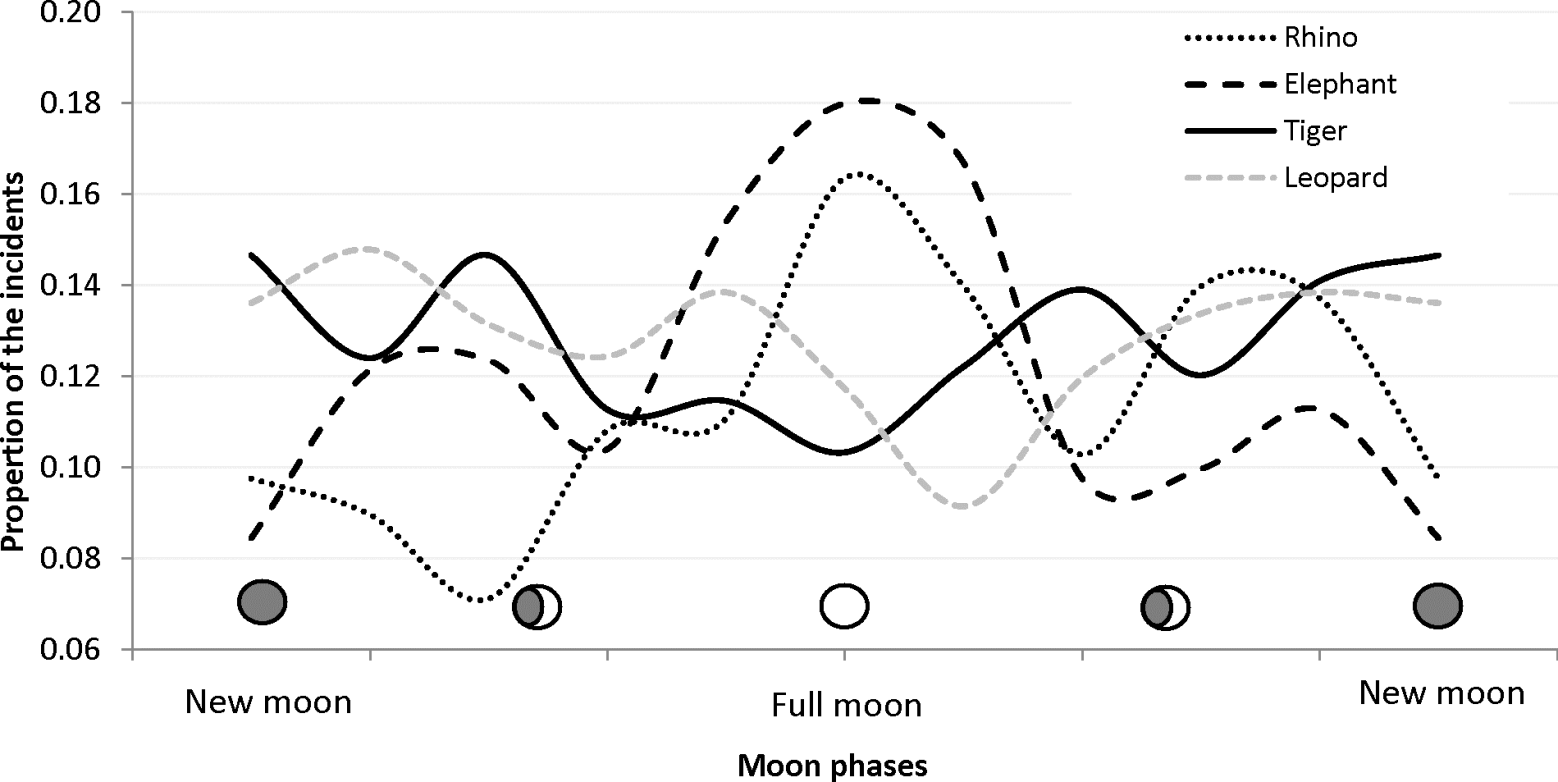
Proportion of the reported human-wildlife interactions with elephant, rhino, tiger and leopard in Chitwan NP between 2001 and 2015 plotted over the lunar days.

A significant difference on the frequency of conflict incidents caused by elephant (χ^2^ = 27.32, df = 5, P = <0.001) and rhino (χ^2^ = 21.54, df = 5, P = <0.001) was observed between the moon phases with more incidents occurring during full moon periods (Fig 3). In contrast to the herbivores, the carnivores had a minimum number of incidents during the full moon period but the relationship was not significant for both tiger (χ^2^ = 7.51, df = 5, P = >0.05) and leopard (χ^2^ = 3.72, df = 5, P = >0.05).

### Human death and injury

**Fig 4:**
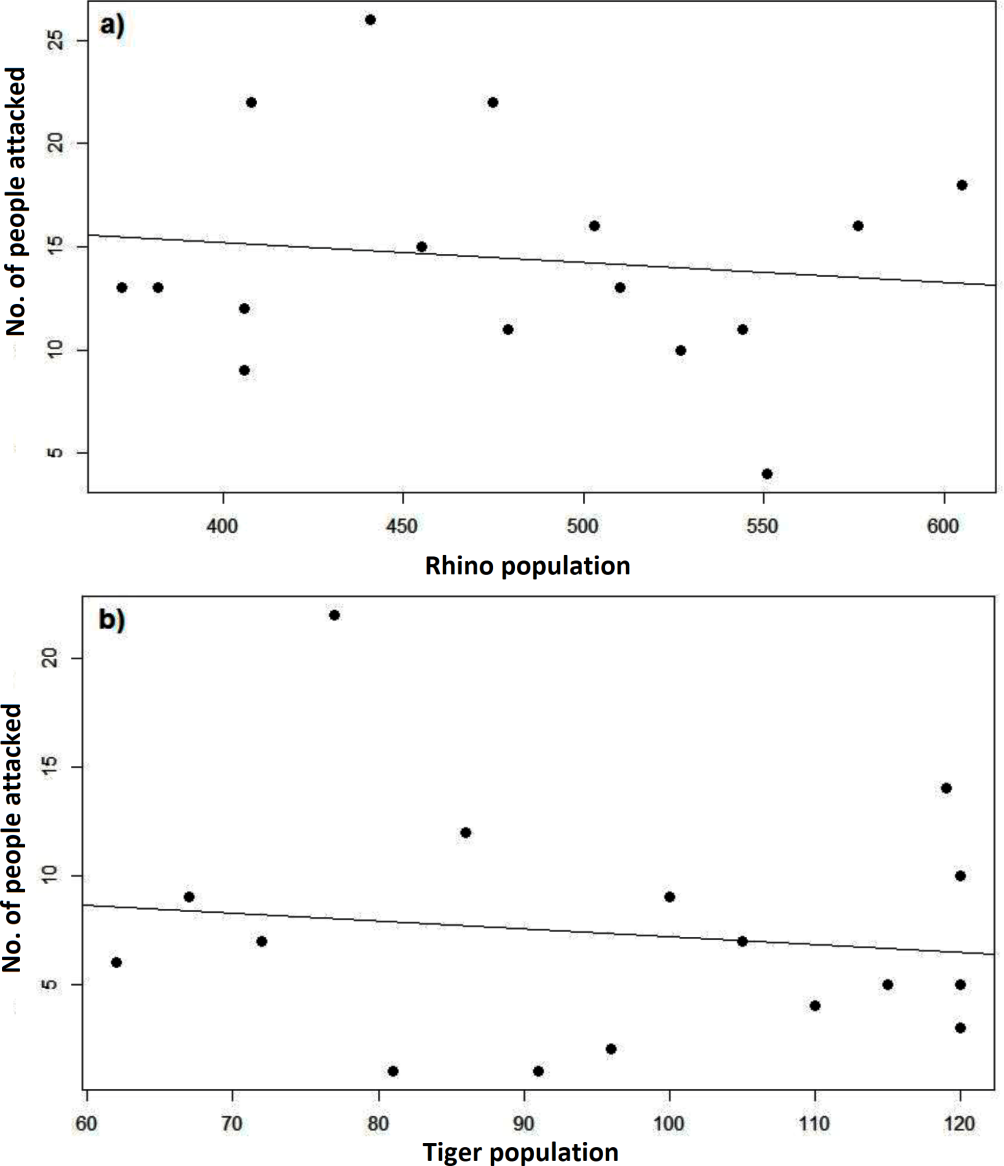
Number of attacks on humans (y-axis) plotted over the population of a) rhino and b) tiger in x-axis.

A total of 732 wildlife attacks with an annual average of 9.3 human death (SD = 5.7) and 31.3 human injury (SD 11.8) were recorded between 1998 and 2016. The linear regression shows a marginal decrease of both human death (- 0.06/year) and human injury (-0.45/year). The annual sum of wildlife attacks on humans varied significantly over the years (χ^2^ = 81.17, df = 17, P = <0.001). Compared to human injuries, a significantly lower (t = 7.1, df = 24.53, p = <0.001) number of the wildlife attacks resulted in human fatalities. The number of attacks by herbivores (rhino, elephant, wild boar, deer) was not significantly different (t = 0.76, df = 30.1, p>0.05) from the number of attacks by carnivores (tiger, leopard and sloth bear). More than two third of the human killings was caused by tiger (38.3%) and rhino (32.1%), but more human injury was caused by rhino (32%, n = 567) and sloth bear (26.1%) compared to tiger (9.9%) and elephants (5.8%) (Table 1). The linear regression analysis did not show a significant influence (P>0.05) of tiger and rhino population trends on frequency of attacks on humans (Fig 4) leading us to rejection our hypothesis.

There was a significant variation in the frequency of wildlife attacks between the different communities (χ^2^ = 305.1, df = 4, P = <0.001). Indigenous and Dalit communities were attacked more frequently whereas ethnic and hill migrant communities were attacked less frequently than expected (Table 2).

**Table 2:** The expected and observed proportion of wildlife attacks on humans of different ethnicity.

Ethnicity of people attacked	Expected proportion (%)	Observed proportion (%)	Deviation from expected (%)
*Hill migrant*	41.7	39.1	-6.8
*Ethnic migrant*	27.8	16.5	-68.3
*Indigenous*	17.3	30.1	42.6
*Dalit*	8.2	11.1	25.5
Others	5.0	3.3	-51.2

**Fig 5:**
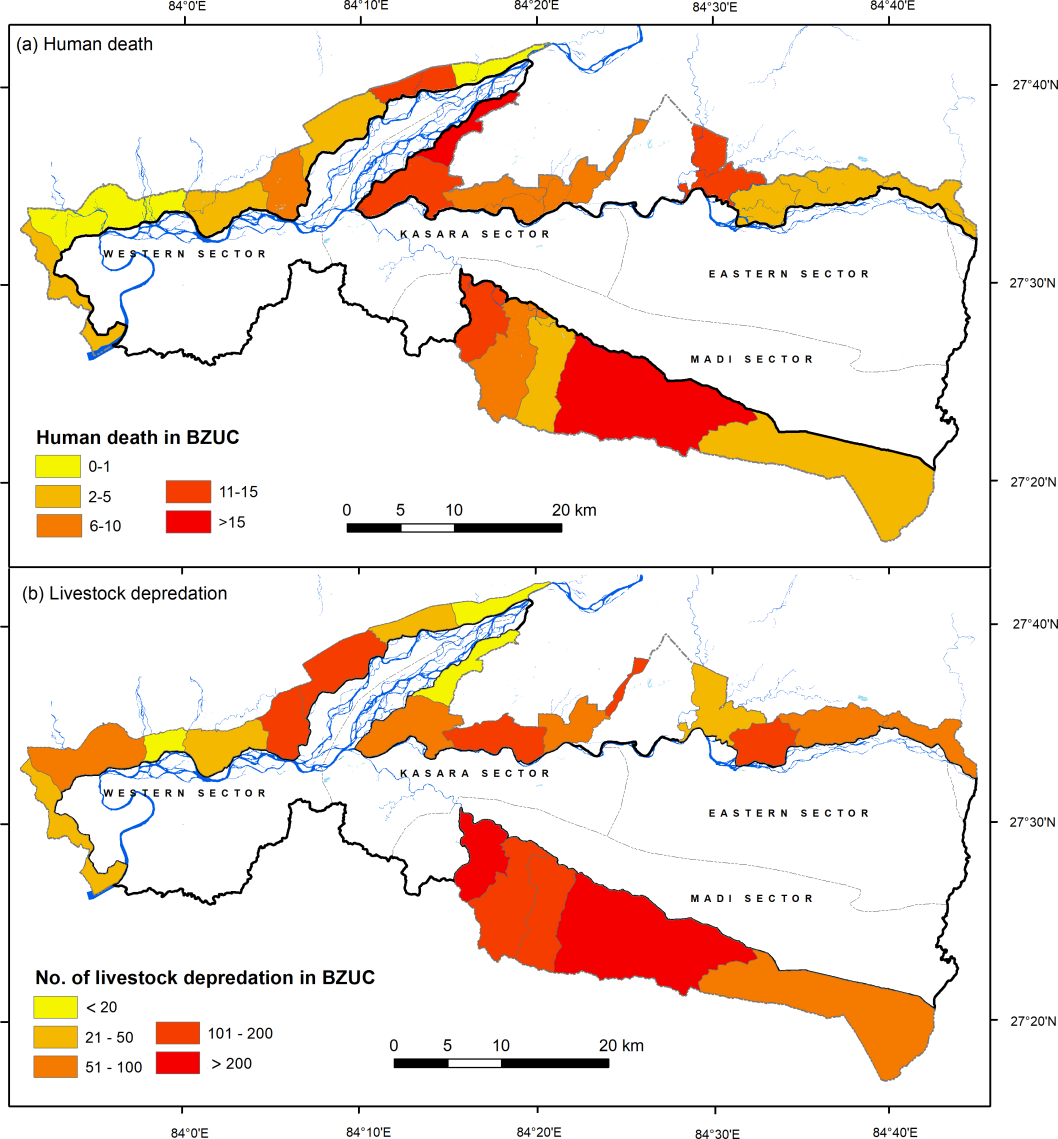
Spatial distribution of a) human killing and b) livestock depredation in buffer zone of Chitwan National Park, Nepal.

Among the BZUCs, a significant difference in the number of attacks on humans (χ^2^ = 257.5, df = 21, P = <0.001) and livestock depredation (χ^2^ = 992.1, df = 21, P = <0.001) was observed (Fig 5). Five of 22 BZUCs of Chitwan recorded > 50% of human deaths and 13 BZUCs reported five or more human deaths in their area. The highest number of human killing (24) was recorded from Ayodhyapuri BZUC in Madi valley (south of the park) followed by Kalabanjar BZUC (18).

### Livestock depredation

**Fig 6:**
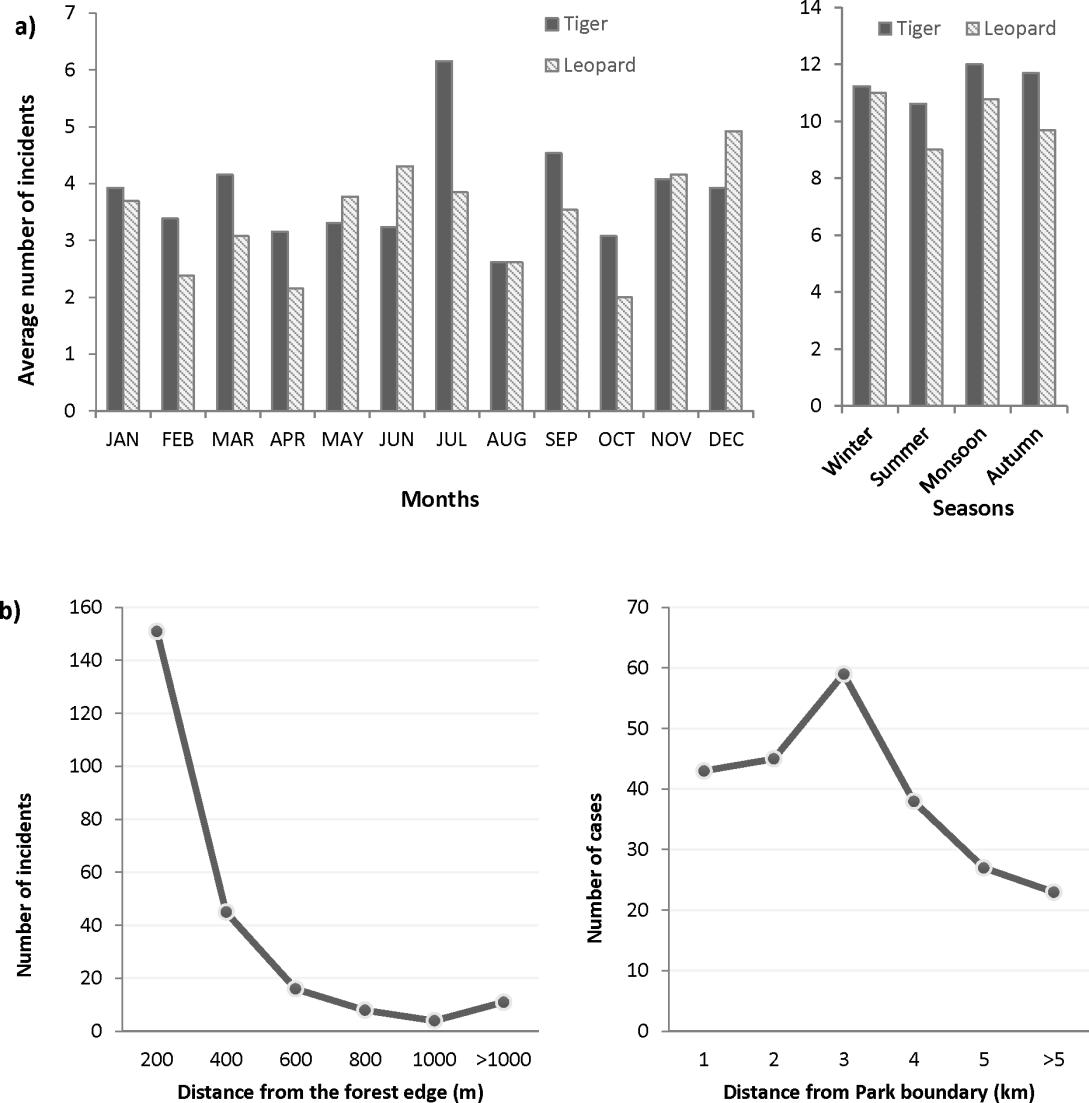
a) Average number of livestock depredation incident per month and season in buffer zone of Chitwan National Park during 1998–2016, b) Number of livestock killed by tiger and rhino in the distance from forest edge and park boundary.

An annual average of 122.94 (SD = 80.97) incidents of livestock depredation were recorded around CNP during the study period. Tiger and leopard caused most (>90%) of the reported livestock depredation (n = 2213). The annual frequency of livestock depredation by tiger was significantly higher (t = 2.2, df = 20, p<0.05) compared to leopards but in recent two years (after 2014) leopards caused more livestock depredation than tigers (Fig 2). The overall trend of livestock depredation between 1998 and 2016 shows an insignificant decline (Fig 2) with a significant variation over the years (χ^2^ = 901.54, df = 17, P = <0.001). A maximum number of livestock were killed during 2002 to 2004 and numbers decreased sharply afterwards. Although some fluctuations were observed, we could not any find a significant difference between the average number of livestock depredation over the months (χ^2^ = 3.87, df = 11, P = 0.97) and seasons (χ^2^ = 0.27, df = 3, P = 0.97) (Fig 6A). We found a significant (p<0.05) negative correlation (Pearson’s correlation coefficient—0.60) between livestock depredation and the number of people on foreign employment over the years in Chitwan district.

There was a significant difference between tiger and leopard’s livestock preference (χ^2^ = 279.58, df = 4, P = <0.001). Tigers killed both medium sized (goat/sheep and pig, 58%) and large sized livestock (buffalo and cow/oxen, 41%) but leopards mostly (>96%) killed smaller sized goat/sheep or pig (Fig 3B).

**Fig 7:**
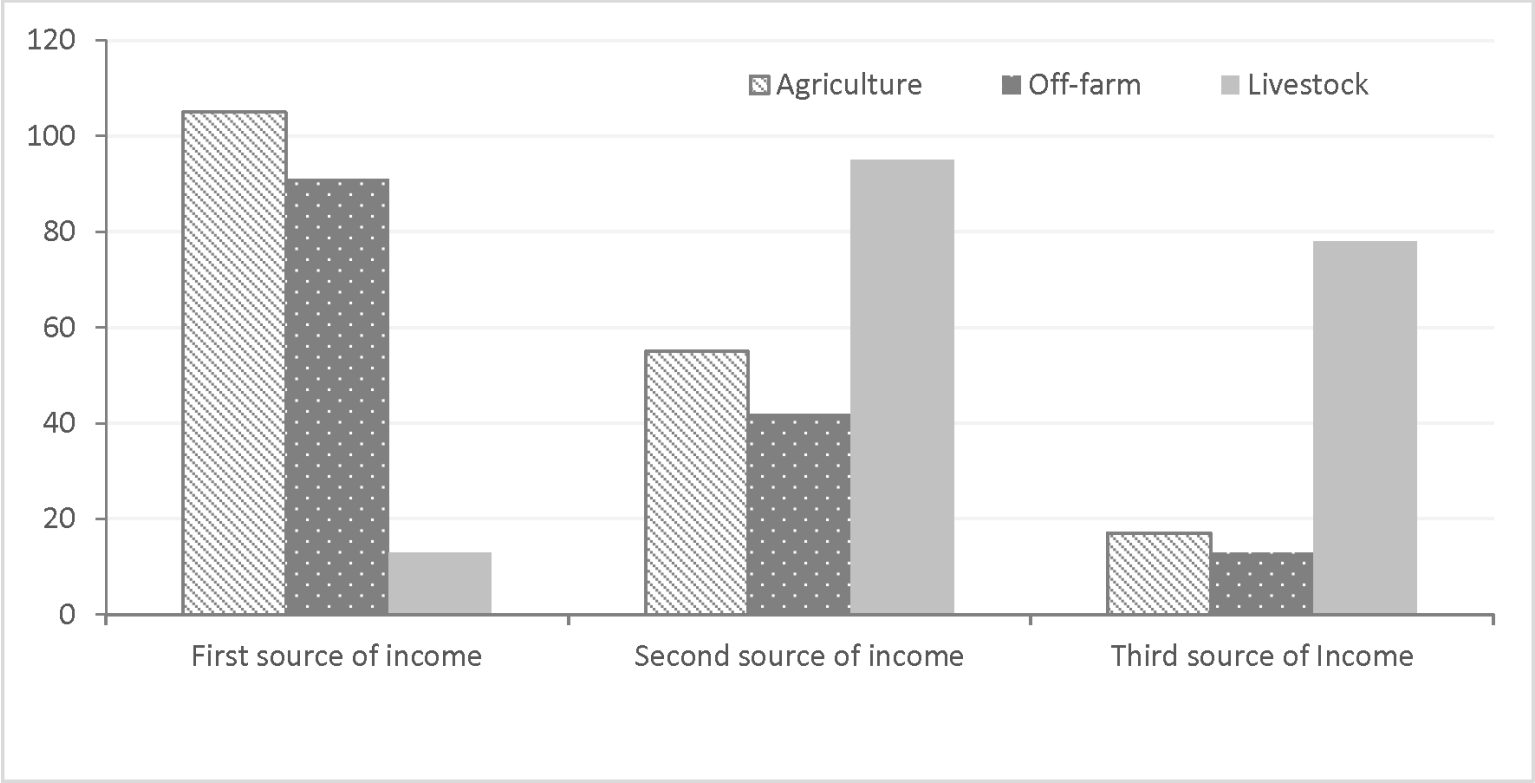
Dependency of livestock depredation victim households on agriculture, livestock and other off-farm activities.

A questionnaire survey with the victim’s households in last five years shows that most of the livestock depredation by carnivores (87.7%, n = 253) were caused inside the stall. The livestock killing occurred mostly (86.8%) in close proximity i.e. <500m distance of the forest village edge (Fig 6B). Both the percentage of households having livestock and the average size of holding (except for the goats) have decreased over the years (Table 3). Livestock contributes an income for 74% of the households and 7% reported it as primary source (Fig 7).

**Table 3:** Percentage of households with livestock, average livestock ownership per household and percentage of households grazing their livestock. Data for 1997 and 2006 obtained from Gurung et al. (2010).

Type of livestock	% Households with livestock			Average per household			% of Households grazing the livestock (2017)
	1997*(n = 354)	2006*(n = 400)	2017(n = 254)	1997*(n = 354)	2006*(n = 400)	2017(n = 254)	
Goat	74	71	**70**	2.80	2.80	**3.27**	11.46
Cattle	57	47	**36**	1.80	1.20	**0.91**	7.51
Buffalo	81	67	**47**	2.50	1.60	**1.02**	11.86
All livestock	94	91	**88**	7.10	5.60	**5.40**	18.04

*The average value comes from Madi valley (Southern buffer zone) of Chitwan National Park.

Most of the carcasses of killed livestock (94.8%, n = 248) were found by the victim families. Three fourth (75.7%, n = 235) of them were buried and 8.9% were consumed within families (6.8%) or neighbors (2.1%). Less than 15% of the carcasses were left and probably consumed by tiger/leopard or scavenger. A majority (60.8%) of the respondents reported the subsequent livestock killing in their locality (village) by the tiger or leopard.

## Discussion

We present the most comprehensive analysis of wildlife attacks on humans and economic loss in buffer zone of Chitwan National Park, Nepal published to date. Livestock depredation was the most frequent among the reported types of losses followed by attacks on humans, crop raiding and property damage. Losses were caused by 12 wildlife species with the maximum number of incidents caused by tiger followed by elephant, leopard and rhino. Although crop raiding by deer and wild boar is widespread, the government guidelines do not provide relief and thus, these remain un-reported (NTNC, unpublished data). Our study shows that the relief claim data can provide a valuable source of information about the human-wildlife conflict.

### Effect of moon phase

Our results partly support the hypothesis that moon phase has an influence on wildlife activity and conflict with humans. We detected significantly higher conflict incidents caused by greater one-horned rhinos and Asian elephants during full moon phase. It is not surprising to find higher conflict incidents of Greater one-horned rhinos during moonlight nights as they are active both day and night with peak during early morning and late afternoon [37]. Our finding of higher incidents of Asian elephants during moon light nights is contrary to Gunn et al. [25] who reported lower incidents of crop raiding by African elephants (*Loxodonta africana)* during full moon nights. Such difference could be due to 1) the behavioral difference between the elephant species, 2) differences in landscape patterns with thick vegetation in Chitwan compared to wide open African savannas and 3) difference in crop guarding practices.

The number of conflict reports of both tiger and leopard was lowest during full moon, the difference was not significant to other phases of the lunar cycle. Packer et al. [22] also documented the higher number of lion attack on humans during dark period in the nights in Tanzania. But higher livestock depredation during the full moon period was reported by Tumenta [23] in Waza National Park, Cameroon. Both tigers and leopards are nocturnal predators [12, 18] causing majority of the attacks on human and livestock in night. There is lack of details on whether they attack during moonlight or dark nights but studies of lions, another nocturnal predator in Africa, shows less success in obtaining wildlife prey during moonlit nights. Our finding was based on conflict records reported by people and our data include date but not time of the incident. This limited our conclusions on the actual effect of moon phase and night luminescence. A detailed study with incident time is required to fully understand the effect of moon phase.

### Human loss & injury

Our report of an average annual of 40.6 wildlife attacks on humans with 9.3 fatalities, is higher than previously reported by Silwal et al. [9]. The total number of wildlife attacks per year could be higher since our data only cover the buffer zone and do not include the incidents when people are illegally entering the core area of the park. Dhungana et al. [26] reported about one fourth of attacks on humans occurred in core area who do not get the relief. Comparing to other protected areas in Nepal, CNP observed the highest rate of human casualties [38–40]. High density of multiple large mammal species (Rhino, Tiger, Gaur, Sloth bear etc.) occur in CNP [3, 28] in close proximity of the human habitation. The Park is narrow and elongated maximizing the interaction zone between humans and wildlife. However, there are other protected areas in South Asia where conflict is more intense like in Sundarbans in Bangladesh where annually 22 human fatalities on tiger attack has been recorded [41].

In spite of an increasing wildlife population in the park and human population in the buffer zone, we did not find the respective increase on the fatalities or injuries from wildlife. This could be attributed to 1) less human-wildlife interaction with reduced dependency of communities on forests, 2) separation of forest and farmlands/settlements by installing electric and mess wire fences along the forest border in the buffer zone with the support of government and NGOs, 3) increased awareness and 4) other preventive measures practiced by communities. The trend of wildlife attacks on humans in Chitwan is more or less stable over the study period but still substantial. Further strengthening of mitigation measures and awareness among communities will contribute in reducing human loss.

There was a difference in the expected and observed rate of attacks by wildlife to members of different ethnic communities. Due to higher dependency on forests for their traditional livelihood practice, frequent interaction of *Terai indigenous* and *Dalit* communities with wildlife resulted more wildlife attacks on them. Both of these groups are underprivileged in society who live in close proximity of the forests [42] and generally have lower economic opportunities for alternative livelihoods. Previous studies from Chitwan show that >80% of the wildlife attacks on humans happen within the 2 km of the park boundary [8–9].

### Livestock depredation

Annual average of 123 livestock killing in CNP is comparable to Bardia National Park (118/year, NTNC unpublished data) in western Nepal but higher than other protected areas. Much higher livestock killing has been reported in some of the Indian Parks (462/year, Kanha National Park) [43]. Grazing restrictions in the core areas of the parks and community managed buffer zone forests have contributed to keep the livestock depredation cases lower compared to Indian parks where free grazing is common [43–44].

Tiger and leopard caused most of the livestock depredation with the highest number of incidents by tigers. However, in recent years (after 2014) leopards caused more losses. Increasing tiger population of Chitwan may have pushed leopards into the fringes of park or in the buffer zone where they kill livestock frequently. A similar observation was reported in Bardia, the other park in Nepal’s Terai [45]. We found a gradual shift of the buffer zone communities towards off-farm based income sources with reduced dependency over agriculture and livestock. Households with livestock as well as average holding have reduced gradually over the years. Most of the households (>80%) practice stall feeding. Out of the grazing households (n = 45) nearly half (46.7%) graze their cattle in community forest, others graze on private land or road-side and other fallow land. Grazing was common until early 2000s [44] in the BZ area but in recent years, stall feeding is facilitated by grazing restrictions, adoption of the improved variety of livestock, the use of commercial livestock feeds and a shortage of labor for grazing. For instance, we found a significant negative correlation between the people on foreign employment and number of livestock killed. We suggest that the increase of foreign employment has reduced dependency on forests as a consequence.

Most of livestock killing occurred at the stall which suggests the need of better husbandry practices with predator-proof livestock corrals. The carcasses were mostly found and buried or consumed by the victim families. This practice is likely to have caused more livestock loss as tiger or leopard could not continue feeding on the carcass for a longer time and they go for another livestock kill. More than 60% of the respondents reported additional livestock killing by the tiger or leopard within couple of weeks’ time in their locality. In the past, before starting relief scheme, the park authorities promoted the burying of a carcass to avoid poisoning in retaliation. Leaving the carcass in safe places in the forest instead of burying it and providing quick relief to the owner will contribute to reduced livestock killing.

### Temporal trend of conflicts

We found an insignificant but decreasing trend of the wildlife attacks on humans and livestock with a significant variation over the years. The reported conflict incidents peaked during 2002–2004. Gurung et al. [20] reported the restoration of community forests in the buffer zone providing refuge habitat for wildlife as a contributing factor. In addition to this, the socio-political situation also contributed in some way to the increased conflict. During 2000–2005, Maoist insurgency peaked in Nepal. During this period, three forth of the guard posts in the park were abandoned and army personnel retracted to larger bases or headquarter leaving the way open for local villagers and poachers to enter more freely [46]. Such disturbance in the park resulted in an increased interaction between people and wildlife leading to more loss of human and livestock. The high disturbance in the core areas of the park might have pushed animals into the fringes for safe refuge.

However, our data did not support the hypothesis that an increase in wildlife populations results in respective increase of conflicts. With reduced poaching [47], wildlife population like rhino and tiger has peaked in recent years in Chitwan whereas higher conflict incidents were recorded during 2002–2004. Highest number of human killing in 2004 can be linked to 3 man-eaters killing >15 people including five persons killed in a single incident [20, 48]. Similarly, an elephant attacks on human peaked in 2012 when a rage elephant was active around Chitwan [9] which attacked >10 people, six of them died. In case of large mammals, not all individuals in wildlife population are equally responsible for human or economic loss but few rage animals make a larger share of the conflict incidents [49]. In addition, the measures of conflict reduction practiced by buffer zone communities and reduced interaction of human-wildlife as mentioned earlier might have kept the conflict incidents in control. Our study has not examined the property damage and crop raiding in detail. We recommend future studies on these aspects to understand and mitigate human-wildlife conflict.

## Conclusion

Our results show that increasing wildlife population is not directly related to the more conflicts. Reduced forest dependency with changing livelihood strategy (reduced grazing, increased off-farm household income), conflict mitigation measures (electric and mess wire fences) and public awareness have largely contributed to reduce the loss from wildlife. Strengthening of the mitigation measures, reducing forest dependency and awareness programs to the vulnerable communities will minimize the conflict. Timely identification and management of problem animals like man-eater tiger and rage elephant will reduce the human killing and injury. Change in livestock husbandry by making more secured or predator-proof corrals especially in forest fringes will reduce the livestock loss.

## Supporting information

S1 File Semi-structured questionnaire used to record the detail information on the livestock depredation cases. (PDF)

S1 Table Amount in USD released in each year for different types of losses by the buffer zone program and Nepal Government over the years. (PDF)
