# Supplementary material for: Spatio-temporal patterns of attacks on human and economic losses from wildlife in Chitwan National Park, Nepal
Source: PLoS One. 2018 Apr 19;13(4):e0195373. doi: 10.1371/journal.pone.0195373 (PMC5908188; doi:10.1371/journal.pone.0195373)
Supplement: S1 File — (PDF) [file pone.0195373.s001.pdf]

S1 File. Semi-structured questionnaire used to record the detail information on the livestock depredation cases.

Form No: [ ]

Date of Incident: Year Month Day **Time**:

1. G.P.S.

House: E N Elev:

Place of incident: E N Elev:

Livestock loss

Which livestock<br><br>(1-Cattle, 2-bufffalo, 3-goat, 4-sheep, 5-pig, 6-other -?)	Killed or injured?<br><br>1-Killed<br><br>2-Injured	Killed by<br><br>(1 - Tiger, 2 - leopard, 3- other , 4-Don't know	How predator was identified?<br><br>(1 - sighting, 2 - track/sign, 4- call/sound, 5 - guess, 6 - other?	Are you confident on identification?<br><br>(1 - Very confident, 2 - not sure, 3 -guess only)

1. Was the carcass found? Yes ( ) No ( ), if yes how far? (m)

2. Was tiger/leopard nearby carcass? Yes ( ) No ( )

3. What was done to carcass?
   a) Left (did nothing) b) buried c) taken out and eaten c) Others ?))

4. Have the tiger/leopard again killed other livestock in the village? Yes ( ) No ( )
   If yes, give details

5. What was the cost of killed livestock?
   a) At that time (NRs) b) What would be price now? (NRs)

6. Have you got relief of the loss ?Yes ( ) No ( ), If yes how much?
   (NRs(
   How long it took to get the relief?

7. Additional information if any
