# Supplementary material for: Spatio-temporal patterns of attacks on human and economic losses from wildlife in Chitwan National Park, Nepal
Source: PLoS One. 2018 Apr 19;13(4):e0195373. doi: 10.1371/journal.pone.0195373 (PMC5908188; doi:10.1371/journal.pone.0195373)
Supplement: S1 Table — (PDF) [file pone.0195373.s002.pdf]

S2 Table. Amount in USD released in each year for different types of losses by the Buffer Zone Program and Nepal Government over the years.

Fiscal year	Human death	Human injury	Livestock loss	House & property	Crop	Total
1998/99	4,059.04	310.42	1,797.86	-	-	6,167.32
1999/00	1,834.19	3,056.33	6,006.93	-	-	10,897.45
2000/01	710.23	1,158.65	2,317.47	-	-	4,186.35
2001/02	3,360.22	2,065.86	4,246.79	422.72	-	10,095.58
2002/03	2,564.10	2,013.14	6,305.21	269.23	-	11,151.68
2003/04	8,274.86	2,789.21	5,583.03	1,065.80	-	17,712.90
2004/05	1,680.33	582.36	3,292.12	295.74	-	5,850.56
2005/06	4,619.76	2,329.44	1,427.68	1,419.49	-	9,796.36
2006/07	1,901.92	3,484.31	2,056.98	1,147.75	-	8,590.95
2007/08	3,076.92	2,872.11	3,394.77	1,430.08	-	10,773.88
2008/09	10,934.54	7,121.97	8,460.83	1,296.94	232.54	28,046.81
2009/10	15,686.27	6,443.69	-	-	-	22,129.96
2010/11	22,222.22	8,775.77	-	-	-	30,997.99
2011/12	21,067.42	6,279.49	-	-	-	27,346.91
2012/13	46,796.66	7,306.87	-	-	-	54,103.53
2013/14	15,839.49	6,899.43	1,821.01	411.83	7,856.39	32,828.15
2014/15	28,130.86	5,081.89	6,286.21	3,478.95	4,777.56	47,755.47
2015/16	24,601.46	19,076.33	7,291.87	2,740.74	11,506.26	65,216.66
Total	217,360.48	87,647.29	60,288.74	13,979.26	24,372.75	403,648.51
